# Primary cultures of mouse small intestinal epithelial cells using the dissociating enzyme type I collagenase and hyaluronidase

**DOI:** 10.1590/1414-431X20175831

**Published:** 2017-04-13

**Authors:** H.J. Ren, C.L. Zhang, R.D. Liu, N. Li, X.G. Li, H.K. Xue, Y. Guo, Z.Q. Wang, J. Cui, L. Ming

**Affiliations:** 1 Department of Clinical Laboratory, the First Affiliated Hospital of Zhengzhou University, Zhengzhou, Henan, China; 2 Key Clinical Laboratory of Henan Province, Zhengzhou, Henan, China; 3 Department of General Surgery, the People's Hospital of Zhengzhou, Zhengzhou, Henan, China; 4 Department of Parasitology, Medical College, Zhengzhou University, Zhengzhou, Henan, China

**Keywords:** Intestinal epithelial cells, Crypt, Isolation, Primary culture, Collagenase I, Hyaluronidase

## Abstract

The epithelium is a highly dynamic system, which plays a crucial role in the homeostasis of the intestinal tract. However, studies on the physiological and pathophysiological functions of intestinal epithelial cells (IECs) have been hampered due to lack of normal epithelial cell models. In the present study, we established a reproducible method for primary culture of mouse IECs, which were isolated from the viable small intestinal crypts of murine fetuses (on embryonic day 19), using type I collagenase and hyaluronidase in a short span of time (≤20 min). With this method, continuously growing mouse IECs, which can be subcultured over a number of passages, were obtained. The obtained cell lines formed a tight cobblestone-like arrangement, displayed long and slender microvilli, expressed characteristic markers (cytokeratin 18 and Notch-1), and generated increasing transepithelial electrical resistance and low paracellular permeability during *in vitro* culture. The cells also had enzymatic activities of alkaline phosphatase and sucrase-isomaltase, and secreted various cytokines (IL-1β, IL-6, IL-8, and monocyte chemoattractant protein-1), responding to the stimulation of *Escherichia coli*. These results show that the primary-cultured mouse IECs obtained by the method established here had the morphological and immunological characteristics of IECs. This culture system can be a beneficial *in vitro* model for studies on mucosal immunology and toxicology.

## Introduction

Intestinal epithelial cells (IECs) play a key role in maintaining the host's homeostasis and in the uptake of nutrients and fluids. They also have the ability to protect the organism from various pathogens and toxins in the intestinal lumen (1). Moreover, some studies have shown that IECs participate actively in the mucosal immune response, by presenting antigen characteristics, secreting various cytokines, and recruiting specific immune cells in response to pathogens and their products (2,3).

The small intestine epithelium can be continuously renewed by cell generation and migration from the crypt stem cells to the differentiated cells at the top of the villus (4). Epithelial cell proliferation, migration, and differentiation are tightly regulated by various mechanisms controlled by a series of growth factors (4). However, the mechanisms that regulate IECs proliferation and differentiation have not been fully elucidated (5). In addition, most serious pathological conditions, such as inflammatory bowel disease, and interactions between pathogens and IECs, will cause alterations of IECs growth and physiological functions. Based on this, it is very important to investigate the physiology and pathophysiology of IECs, especially for studies on intestinal physiology, intestinal immunology, and cancer genesis. However, studies at the cellular and molecular levels in the natural hosts are impossible for ethical and financial reasons, in most cases. Thus, an *in vitro* IEC model similar to the *in situ* epithelium is needed. Among the most used models, the ones with primary cultured or immortal cells are particularly favored today (6).

Immortal IEC cell lines have been established from human colon cancers (Caco-2, HT-29, HCT8, T84) (7–9) and from rat and chick embryos by spontaneous transformation (10). Although they can undergo a complete intestinal-like program of differentiation (11), the applications of human colon cancer cell lines have been limited by their cancerous characteristics. Continuous IEC cell lines have also been derived from human, rats, bovine, and pigs, after immortalization by oncogene transfection (12–14). The transgenic cell lines have advantages over primary cultures due to their serially-passaged characteristic. However, it is evident that part of their original functions can be changed because of immortalization (15). For instance, primary IECs derived from adult mice intestines expressed MHC II molecules and presented antigen to T cells without induction of interferon (IFN)-γ (16), but some mouse IEC lines did not, such as MODE-K (17).

In light of these limitations, continuously growing cultures of primary IECs would be very useful. In recent years, many efforts have been made to culture primary IECs, and several techniques have been described (14,18). Since *in vitro* survival time of these isolated IECs is very limited, and extensive cell death is observed within a few hours after plating, obtaining short- or long-term cultures of IECs is difficult. The recent discovery of crypt-derived primary tissue culture allows the analysis of viable primary IECs from variable sources (19). Unfortunately, tissue cultures are time-consuming and expensive, and they are impractical for large-scale analyses. Consequently, it is necessary to explore methods for propagating freshly isolated IECs within a short term, allowing reproducible quantitative studies.

In our previous studies, various mechanical and/or enzymatic methods have been tested, and it was found for the first time that the combination of type I collagenase and hyaluronidase considerably shortened the time of isolation and improved the yield of growing non-mesenchymal epithelial cells. Moreover, we also found that primary IECs (at passage 8) obtained using this method could be invaded by the intestinal parasite *Trichinella spiralis* (20). In the present study, the morphological and biological characterization of the established mouse IEC line, which was derived from the fetal small intestinal crypts isolated using type I collagenase and hyaluronidase, was further investigated. This novel method provides a versatile tool to generate stable IEC lines for functional and structural analyses.

## Material and Methods

### Experimental animals

Male and female BALB/c mice, 6-8 weeks of age, were purchased from the Experimental Animal Center of Henan province (China), and bred in plastic micro-isolator cages. In all the experiments, mice were sacrificed by cervical dislocation. All animal procedures were reviewed and approved by the Animal Care and Use Committee of Zhengzhou University (Permission No. SYXK 2011-0001).

### Crypt isolation procedure

The culture medium used was Dulbecco's modified Eagle's medium (DMEM; Gibco, USA) supplemented with glutamine (4 mM; Sigma, USA), sodium pyruvate (1 mM; Sigma), Hepes (20 mM; Sigma), penicillin (100 U/mL; Amresco, USA), streptomycin (100 µg/mL; Amresco), bovine insulin (0.1 U/mL; Sigma), and 10% (or 5%) fetal bovine serum (FBS; Gibco), hereafter referred to as the complete DMEM.

BALB/c fetuses were removed on embryonic day 19 (E19) by cesarean section and were kept in ice-cold phosphate-buffered saline (PBS) (21). The mesentery was discarded, and then the small intestines were gently and rapidly removed from the abdominal cavity of the fetuses, opened longitudinally, and immersed in PBS. The intestines were minced into 1-mm long fragments with sharp scissors. The fragments were then transferred into a 15 mL centrifuge tube, washed five times in PBS, and subsequently incubated at 37°C under agitation for 20 min in the presence of type I collagenase (200 U/mL; Sigma, USA) and hyaluronidase (100 U/mL; Sigma, USA). Meanwhile, thermolysin (50 μg/mL; Sigma, USA) was also used to digest intestine tissues for comparison. Following gentle dissociation by a pipette, incubation solutions were carefully removed and centrifuged at 100 *g* for 5 min at 4°C. The remaining pellets were washed with DMEM containing 2% FBS and 2% sorbitol (Amresco), and the pellets containing the purified crypt fraction were collected by centrifugation at 250 *g* for 5 min at room temperature (RT). The isolated crypts (pellet) were then resuspended in complete DMEM (10% FBS) and crypt number was estimated.

### Cell culture

The crypts were then seeded on 25-cm^2^ plastic culture flasks (Corning, USA). The optimal seeding density for the isolated crypts was about 200 crypts per cm^2^. Then, the medium with the non-adhering cells was recovered after 90-min culture and plated into a new plastic culture flask. After 24 h the medium with nonattached cells was removed. Following the addition of fresh complete DMEM (5% FBS), the attached crypts were counted. Plating efficiency was calculated using the following equation: Plating efficiency (%) = 100 × (number of seeded crypts - number of attached crypts)/number of seeded cells. In order to enhance the attachment of the crypts, 10% FBS was added to the medium, and after that only 5% FBS was used until single cell clones were obtained. The medium was changed every 48 h and confluence was reached within approximately 8-9 days. Subcultures were performed after trypsinization (0.5% trypsin, 0.54 mM EDTA in PBS, at 23°C for 5 min).

### Purification of intestinal epithelial cells

In primary culture, fibroblasts usually mixed with IECs or surrounded the crypts, which may grow either in groups or scattered. To ensure the purity of the IEC culture, two combined techniques were used to eliminate contaminating fibroblasts: preplating and differential trypsinization with 0.25% trypsin (Amresco) (22). As IECs and fibroblasts have different tolerance to trypsin, scattered fibroblasts were detached from the culture flask wall first while the IECs remained attached when treated with trypsin. In this study, the cells were rinsed twice with PBS, followed by digestion with trypsin (2 mL) at 37°C for 1 min. Then fibroblasts contracted and became round, while no obvious changes were observed in IECs. At that time, digestion was terminated by the addition of complete DMEM, and the flask wall was washed with PBS repeatedly. A large amount of fibroblasts were then washed away, but the IECs were still on the flask wall. On the other hand, fibroblasts can attach to the wall much faster than IECs. After cell suspension was incubated in the flask for 90 min, the medium with the non-adhering cells (mainly IECs) was transferred into a new flask, followed by replacement of the medium 24 h later.

The first three passages of primary cultures were performed to remove fibroblasts using the combined methods described above, and after three passages few fibroblasts could be found. The IECs of the primary cultures were then cloned using the limiting dilution method in order to establish the mouse IEC line. Cells were diluted to 40 cells/mL in 10% FBS-DMEM, and 100 μL was aliquoted into each well of 96-well plates. Each well was microscopically observed for cell growth and monoclonal expansion at day 3 after plating. The wells with a single colony of rapidly growing epithelial-like cells were obtained. The colonies that grew to confluence were transferred to 6-well plates. When confluent, cultures were transferred to 25-cm^2^ culture flasks. Cell populations were expanded after the cloning and transferred to mass culture in cell flasks. The first confluent culture in a 25-cm^2^ culture flask obtained from each original colony was named “passage No. 1”. The IECs were frozen in liquid nitrogen in 1-mL aliquots (2-3×10^6^ cells/mL in DMEM with 10% dimethyl sulfoxide and 20% FBS).

### Cell proliferation and growth curve

Cell growth was measured by MTT [3-(4,5-dimethylthiazoly-2-yl)-2,5-diphenyltetrazolium bromide] assay (23). Briefly, IECs were harvested conventionally by digestion, seeded on 96-well plates (5000 cells/well) in quadruplicate, and incubated at 37°C in a humidified atmosphere of 5% CO_2_ and 95% air. At various points in time, cells were incubated with 20 μL of MTT solution (5 mg/mL; Sigma) at 37°C for 4 h, followed by solubilization with 100 μL of 100% dimethyl sulfoxide (Sigma) at 37°C for 10 min. The absorbance of each well was measured with a microplate reader (Bio-Tek, USA) at a wavelength of 570 nm. The absorbance is proportional to the viable cell number. All experiments were performed in triplicate.

### Hematoxylin and eosin (HE) and immunofluorescence (IF) staining of IECs

Monolayers of mouse IECs were grown on glass coverslips in 6-well plates. After rinsing with PBS, the cells were fixed with ice-cold acetone for 10 min, and then HE and IF staining were performed. The major steps of IF staining were follows. The cells were permeabilized with PBS containing 0.1% Triton X-100 (Sigma) and blocked using 3% bovine serum albumin (BSA) in PBS. The primary antibodies (Table 1) were diluted in 1% BSA in PBS and incubated with the IECs at 37°C for 1 h, followed by three washes in PBS for 5 min each. The cells were then incubated with FITC-conjugated secondary antibodies (Table 1) for 30 min at RT and rinsed again in PBS. Finally, the coverslips were mounted on glass slides and examined under a fluorescence microscope (Olympus, Japan). In negative control groups, the primary antibodies were replaced with PBS.

**Table 1:**
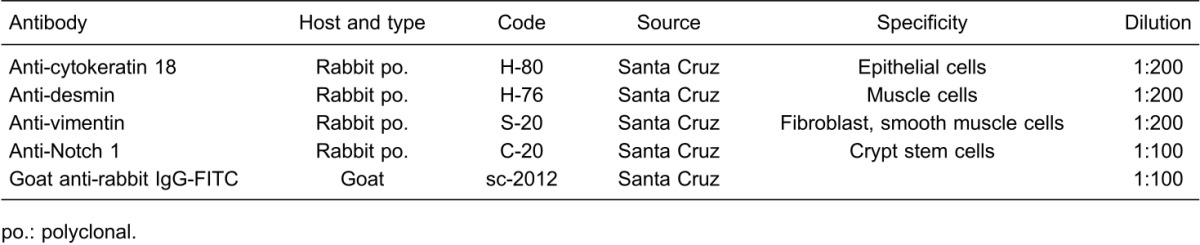
Antibodies used in cultured mouse intestinal epithelial cells.

### Scanning electron microscopy (SEM)

Cells grown on glass coverslips were used for SEM analysis as described previously (24). Briefly, cells were fixed with 4% glutaraldehyde (Sigma) in 0.1 M cacodylate buffer, pH 7.4, for 4 h at RT, followed by post-fixation in 2% OsO_4_ (Amresco) in 0.1 M cacodylate buffer for 1 h. The cells were washed in distilled water, dehydrated in a graded series of ethanol (Amresco) and then substituted with acetone (Amresco), dried by sublimation in Peldri II (Plano GmbH, Germany), sputter-coated with gold, and at last observed by SEM (JSM-7500F; JEOL, Japan).

### Transmission electron microscopy (TEM)

Mouse IECs grown in cell flasks were collected by gentle scraping and fixed for 4 h at 4°C in 4% glutaraldehyde in 0.1 M cacodylate buffer, pH 7.4, followed by post-fixation in 2% OsO_4_ in 0.1 M cacodylate buffer. The cells were then dehydrated through a series of graded alcohols and embedded in Epon 812 resin (Shell Chemical, England). Polymerization of the resin was performed at 65°C overnight. Sections were cut on an Ultracut E (Leica, Germany), stained with uranyl acetate (4 in 50% ethanol) for 15 min and lead citrate (3%; SPI, USA) for 20 min, and examined in a Hitachi 7500 electron microscope (Japan).

### Assessment of cell monolayer integrity

The integrity of the cell monolayer was determined by measurement of paracellular permeability and transepithelial electrical resistance (TEER) (18). Briefly, 1.5×10^6^ cells were seeded on transwell cell culture inserts (1.12 cm^2^/well, Corning, USA) with a pore size of 0.4 μm. The TEER was measured with a volt/ohm meter (EVOM; World Precision Instruments, USA) for a period of 10 days with renewal of the culture medium every other day. The TEER value was calculated using the following equation: (cell well TEER - blank well TEER)×well area size (Ω·cm^2^). Paracellular permeability was determined by 10 kDa FITC-dextran (Sigma). Five milliliters of 10% FBSD MEM containing FITC-dextran (final concentration of 1.0 mg/mL) was added to the apical surface of the monolayer. After 3 h of incubation at 37°C, both apical and basal medium were collected for fluorescence assay using a Fluoroskan Ascent (Thermo, USA). Paracellular permeability was quantified by the apical-to-basal flux rates of FITC-dextran. All experiments were performed in triplicate.

### Enzymatic activities

Activities of the brush border enzymes alkaline phosphatase (ALP) and sucrase-isomaltase (SI) were determined according to the method described previously (25). Briefly, mouse IECs were collected on days 2, 4, 6, and 8 of culture, and enzymatic activity was measured in triplicate using spectrophotometric methods. Enzymatic activity is reported as nmol·mg protein^-1^·min^-1^, assessed according to the method of Bradford (1976) (26).

### *In vitro* inflammation model

The mouse IECs were cultured in 24-well plates at a density of 2×10^4^ cells/cm^2^. When the cells reached confluence, they were incubated with 10^7^ CFU of *Escherichia coli* (ATCC25922) per mL for 5 h at 37°C. The cells then were washed twice in PBS, and further incubated with culture medium containing gentamicin (50 μg/mL; Sigma) at 37°C for 4 h to kill the remaining extracellular bacteria. After a total of 9 h of incubation, the culture supernatants were collected and filtered by a 0.22-μm pore-size filter (Corning). The levels of interleukin (IL)-1β, IL-6, IL-8, and monocyte chemoattractant protein-1 (MCP-1) in the supernatants were determined using ELISA kits (R&D Systems, USA). Unstimulated cells were used as controls for each experiment. All samples were tested in triplicate.

### Statistical analysis

Data are reported as means±SD. The results were analyzed by one-way factorial ANOVA (LSD test) using SPSS version 17.0 software (SPSS Inc., USA). A P value of <0.05 was considered to be statistically significant.

## Results

### Acquisition and purification of mouse IECs

**Figure 1:**
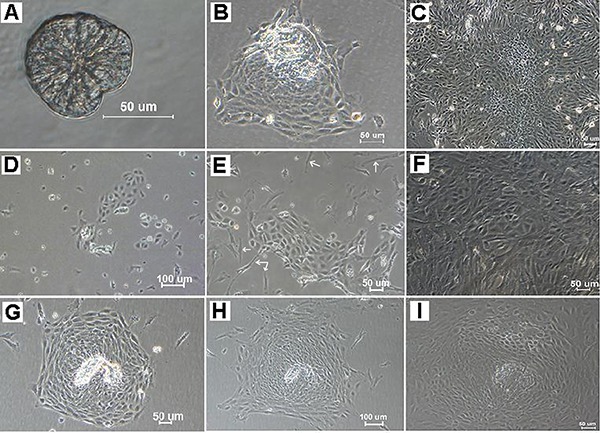
Proliferation of primary-cultured fetal mouse intestinal epithelial cells obtained using different enzymatic methods. Crypts were isolated from fetal mouse intestines using collagenase I/hyaluronidase digestion (*A*). Proliferative epithelial cells gradually migrated out around the crypts within 24 h (*B*), formed large colonies after 5 days (*C)*, and continued to spread extensively before confluence was reached. While trypsin digestion yielded mostly single cells, only part of epithelial cells were adhesive and grew slowly (*D*). Epithelial cells were mixed with fibroblasts (white arrows), which grew either in groups or scattered (*E*, *F*). In addition, thermolysin also appeared to give a few crypts, although mostly in single cells. Proliferative epithelial cells migrated outward after 24 h, and spread extensively and formed colonies after culturing for 2 to 6 days (*G*, *H*). However, the colonies later stopped expanding, and part of those cells began to degenerate after 10 days (*I*).

As a first step in the generation of viable mouse IECs, we have tested several different enzymatic digestions for the isolation of crypts. The results are showed in Table 2 and Figure 1. Although all of these methods yielded IECs, combined digestion of collagenase I and hyaluronidase appeared to give the greatest number of viable crypts in the shortest time (Figure 1A); in most cases viability studies showed that 95% of the cells in the isolated crypts were viable, based on trypan blue exclusion. The plating efficiency of the crypts was 76±10%. Crypts rapidly attached to the flasks, and a few cells gradually migrated out around the crypts within 24 h in culture (Figure 1B). Then, the cells continued to divide extensively after culturing for up to 9 days (Figure 1C) before they reached confluence.

**Table 2:**
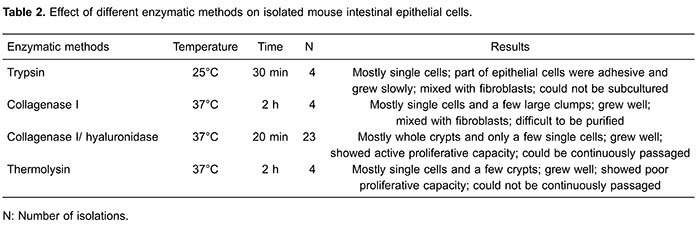
Effect of different enzymatic methods on isolated mouse intestinal epithelial cells.

**Figure 2:**
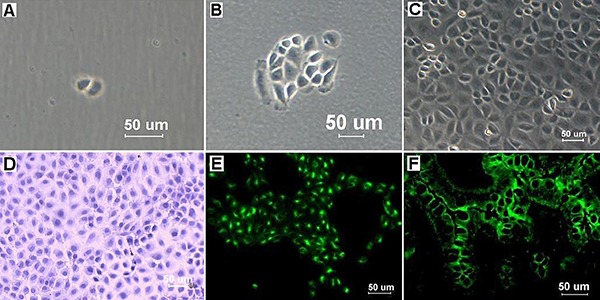
Preparation of cell clones and morphology characterization of mouse intestinal epithelial cells (IECs). Mouse IEC clones were obtained by limiting dilution, and light micrographs showed cell clones at 2 days (*A*) and 5 days (*B*) after seeding. The morphology of mouse IECs at the 35th passage is shown in (*C*). Hematoxylin and eosin staining showed that mouse IECs formed a cobblestone monolayer, and each cell was polygonal and flattened with a large, oval nucleus (*D*). The mouse IECs were strongly positive for cytokeratin 18 (*E*), and the mouse intestinal tissues also reacted with the cytokeratin 18 antibodies as the positive control (*F*).

The contamination of most fibroblasts was eliminated during the purification process of the cultures. By discarding the attached cells after preplating for 2 h or by removing the detached cells after 1 min of trypsinization, few fibroblasts were observed at passage 3. Then, viable IECs were cloned by limiting dilution as described above. The well in which a single epithelial-like cell existed was screened out after 12 h of culture. A total of 5 clones were obtained in our experiments. Cell division of these clones was observed within 48 h of culture (Figure 2A), and cell colonies were formed after 5 days (Figure 2B). When about 90% of confluence was reached in a well after 22 days, cultures were subcultured sequentially into 24-well plates, 6-well plates, and finally T-25 flasks.

### Morphological characteristics and growth curve of mouse IECs

Structural characterization of mouse IECs was observed under a light microscope. Colonies showed the characteristic morphology of epithelial cells, such as an adherent monolayer, a cobblestone-like arrangement and a tightly packed pattern (Figure 2C). Meanwhile, HE staining showed that each cell appeared polygonal and flattened, with a large, round nucleus, typical features of normal epithelial cells (Figure 2D). These primary mouse IECs obtained in our study have been maintained for 56 passages, and no obvious morphological change has been observed. The results of karyotype analysis showed that the IECs possess a normal mouse karyotype, even at passage 48. The modal number, 40, corresponds to the diploid state. No metaphases with a higher chromosome number were observed.

**Figure 3:**
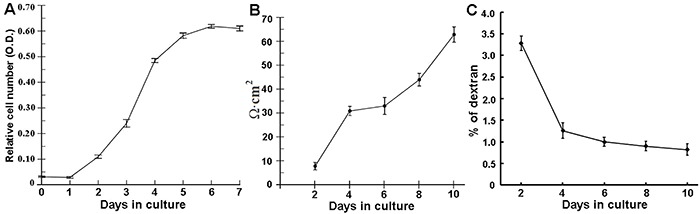
The cell proliferation log phase started after the lag phase of about 2 days with a sharper inclination (*A*). Cells were grown on the apical chamber of transwell-inserts. Transepithelial electrical resistance TEER was measured using an EVOM epithelial volt/ohm meter (*B*). Paracellular permeability was quantified by apical-to-basal flux of 10 kDa FITC-dextran (*C*). All samples were tested in triplicate. Data are reported as means±SD.

Cell proliferation was measured by the MTT assay, and the growth curve was plotted at the indicated time points (Figure 3A). The analysis of the growth curve showed that after an initial lag phase of about 48 h, the cells entered the log phase (96 h), and then the cell growth reached the plateau phase.

### immunofluorescence staining of IECs

Cells were tested at the 27-30th passage for the phenotype of mouse IECs. The expression of the epithelial cell marker cytokeratin 18 (CK18), were examined in cytoplasm of the IECs by IF, indicating the epithelial characteristic of these cells (Figure 2E). Notch-1 is a protein expressed in human and murine intestinal stem cells (27). IF staining showed that Notch-1 was expressed in the membrane and cytoplasm of some IECs, indicating that crypt stem cells, were mainly located in the center or at the border of the cell colonies, from which the well-formed monolayer actually originated. In contrast, desmin, a marker for muscle cells, was not detected in the IECs. The protein vimentin, an intermediate filament protein expressed in fibroblasts and connective tissue, was also not detected in the IECs. The mouse intestinal tissues reacted with the CK18 antibodies and were used as the positive control (Figure 2F), while no green fluorescence was observed in the negative control.

### Ultrastructural characteristics of mouse IECs

**Figure 4:**
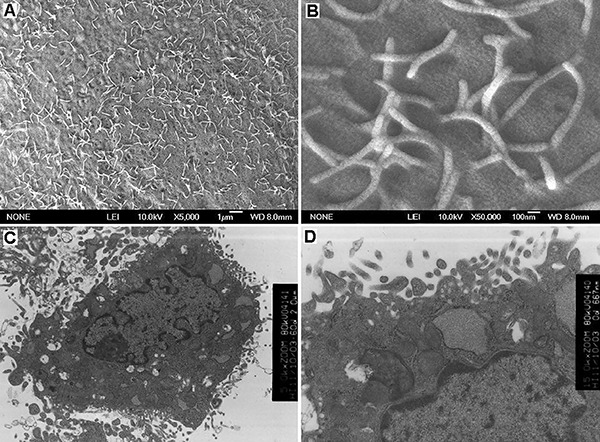
Ultrastructural characteristics of mouse intestinal epithelial cells (IECs). Scanning electron microscopy (SEM) showed that abundant microvilli were observed on the apical surface of the IECs (*A*, *B*). Transmission electron microscopy (TEM) of a cross-section demonstrated that IECs nucleus was large and irregular, showing large prominent nucleoli (*C*). TEM also revealed abundant microvilli on the surface of the cells (*D*).

SEM was used to further observe the surface ultrastructure of the IECs. The epithelial-like appearance of these cells is clearly seen in Figure 4. Long, slender microvilli (Figure 4A and B) were numerous on the cell surface. TEM showed that the flattened cells in culture had a number of cytological typical features of intestinal epithelial cells. The nucleus of mouse IECs was large and irregular, showing one large prominent nucleoli (Figure 4C). Mitochondria, rough endoplasmic reticulum, free ribosomes, and other kinds of organelles were abundant in the cytoplasm. The mitochondria had clear cristae. TEM also revealed abundant microvilli on the surface of the cells (Figure 4C and D).

### Assessment of tight junction of mouse IECs

Mouse IECs were cultured on transwell-inserts for 10 days, and the functional integrity of cell monolayer was investigated by TEER and paracellular permeability every 2 days. TEER of mouse cells increased gradually from 7 to 63 Ω/cm^2^ with culture time (Figure 3B). The transport of FITC-dextran on day 4 of culture was not significantly different from that on day 10 of culture (P>0.05). The permeability of FITC-dextran from the apical to basal aspect of mouse IEC monolayers decreased from about 3.3 to 0.8% during the 10 days of experiment (Figure 3C). These results indicate that mouse IECs can form a monolayer with tight junctions within 4 days after seeding.

### Brush border enzymes activity

**Figure 5:**
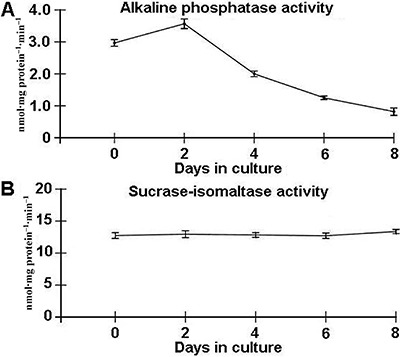
Detection of intestinal brush border enzymes activity in mouse intestinal epithelial cells (IECs). Alkaline phosphatase (*A*) and sucrase-isomaltase (*B*) specific activity in mouse IECs were measured during 8 days of culture. All samples were tested in triplicate. Activity values are reported as means±SD.

The level of activity of two well-known intestinal brush border enzymes ALP and SI were measured at several culture passages. ALP activity was analyzed in all samples and was highest on day 2, decreasing over time, and then maintaining a low level on day 8 (Figure 5A). SI activity was also detected on days 2, 4, 6, and 8; however, it showed no significant variation during cell culture (Figure 5B).

### *In vitro* inflammation assay

**Figure 6:**
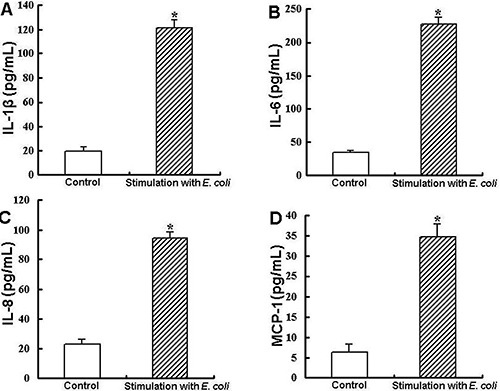
Secretion of cytokines by mouse intestinal epithelial cells (IECs) in response to *E. coli*. Data are reported as means±SD. P<0.05, compared to control (one-way ANOVA).

The responses of primary fetal mouse IECs to *E. coli*, a representative gram-negative bacterium, were measured. IL-1β, IL-6, IL-8, and MCP-1 could be detected in the culture supernatant of the mouse IECs without any stimulation (Figure 6). The treatment with *E. coli* caused the significant increase of cytokines IL-1β, IL-6, and IL-8 (Figure 6A-C) in the supernatant. Moreover, the IECs treated with *E. coli* secreted higher amounts of the chemokine MCP-1 than unstimulated cells (Figure 6D).

## Discussion

The availability of physiologically relevant *in vitro* models is the major limitation in basic and applied research. For *in vitro* studies, primary cells cultures and immortalized cell lines are currently the two possibilities. Primary cells closely mimic the *in* vivo microenvironment as compared to cell lines (18). However, it is difficult to obtain differentiated epithelial cells from normal mammalian intestine *in vitro*. The present study showed that continuously growing IECs can be obtained easily from fetal mouse intestine by a reproducible method. The essential step in this procedure was the application of type I collagenase and hyaluronidase for the rapid isolation of small intestinal crypts. Collagenase, which was used mostly for tissue dissociation, released many intestinal crypts, but this procedure took much longer (>2 h) and gave rise to cultures consisting of many cell types. A combination of type I collagenase and hyaluronidase accelerated the isolation of crypts, and maintained the integrity and cell vitality of the crypts. Our results showed that the plating efficiency of the crypts isolated by the combined enzymes was kept at a high level.

Considering fibroblast contamination, in this study different protocols of enzymatic digestion were tested in order to optimize the isolation of intestinal crypts. Tryptic digestion could release a number of single IECs from mouse intestinal tissues, but only part of them remained viable and attached after 24 h of culture. Furthermore, fibroblasts mixed with IECs were overgrown so that it was very difficult to purify IECs, and the subculture could not be established. Thermolysin has been used in the past to isolate several kinds of cells, such as human IECs (28). Thermolysin seemed to favor the release of clumps of both proliferative and non-proliferative IECs (28). Although in the present study a few crypts were released using thermolysin and attached well, they seemed to be non-proliferative epithelial colonies. These cells gradually spread extensively for up to 6 days then began to degenerate. The reason why the result was inconsistent with the arguments in the literature is not known. We speculated it might be related with different tissue origin and the time- and site-specific susceptibility. We found that a combination of collagenase and hyaluronidase was more effective in the release of many crypts within a short time, greatly reducing the isolation time (≤20 min) and consequent fibroblast contamination. It is worth mentioning that in this study sorbitol was also used to purify crypts. The crypts in DMEM containing 2% FBS and 2% sorbitol could be concentrated to a pellet by low speed centrifugation, but single cells in the supernatant was the final result. Hence, the enzymatic digestion of collagenase I and hyaluronidase, and the use of sorbitol ensured the isolation of viable and pure crypts. In addition, fragmentation and slight shaking of intestinal tissues were also critical steps in obtaining crypts. The small intestines should be finely cut into small pieces rather than mashed. During enzymatic digestion, overly strong shaking should be avoided.

During primary epithelial cell cultures, one of the most common problems is fibroblast contamination, which has huge implications. Although this problem is not usually reflected in the literature, it causes failure in a number of primary culture experiments, meaning a waste of resources (29). Moreover, the influence of the cocultured fibroblasts until they are removed can change some results (30). Studies also demonstrated recently that the effects of coculture can last for several weeks after epithelial cell isolation (31). Usually, fibroblasts can be removed from cultures by scraping, but this procedure is very subjective, and it is used only when fibroblast contamination can be observed with the naked eye. In the present study, two combined techniques were tested to eliminate fibroblasts: preplating and differential trypsinization. The procedure was not based on morphologic characteristics, but on the inherent differential properties of epithelial cells and fibroblasts. This made the procedures more objective and the reliability of the outcomes significantly improved. In addition, in the first 24 h, 10% FBS was used to optimize the attachment rate of the crypts, and subsequently the concentration of FBS was decreased to 5%, which inhibited the overgrowth of the remaining fibroblasts. Through the measures above, fibroblast contamination could be almost entirely eliminated in the primary culture.

The epithelial origin and nature of mouse IECs were confirmed by morphological identification and IF. Light microscope and SEM observations revealed that mouse IECs possessed cobblestone morphology and long slender microvilli on their apical surface, demonstrating a certain differentiation status of the IECs. Nevertheless, the density of microvilli of IECs was lower than some other cell lines, such as Caco-2 with a well-developed brush border (32). Microvilli with a low density were also found on primary IECs from the neonatal dog (32). This difference might be due to different species or the fetal origin, and it might also be possible that they are at a relatively undifferentiated stage. So far, the exact reason remains unclear. In addition, ultrastructural investigation revealed that the cultured cells exhibited structures typical of IECs, such as apical microvilli, numerous mitochondria, a well-developed endoplasmic reticulum, and an extensive Golgi complex.

Mouse IECs were strongly immunopositive for the specific anti-cytokeratin 18 antibody, which confirmed that mouse IECs are, in fact, of epithelial origin (33). In contrast, mouse IECs did not express desmin nor vimentin, which are cytoskeletal proteins expressed by non-epithelial cells such as muscle cells and fibroblasts, respectively. These results showed the intestinal epithelial nature of mouse IECs. Meanwhile, part of mouse IECs expressed Notch-1, a marker for intestinal crypt cells (27), indicating that these serially-passaged IECs retained the potential ability of proliferation. Furthermore, functional differentiation of mouse IECs was determined by detection of brush border ALP and SI activity. ALP and SI are considered to be two differentiation markers for mature enterocytes (34). The established IEC line had ALP and SI activity, strongly suggesting that these cells, in spite of an embryonic origin, differentiated to mature enterocytes to some degree; besides, enterocytes have high ALP activity, while goblet cells and M cells do not. Thus, ALP is also used to identify enterocytes, mucus-secreting goblet cells, and M cells (35). The formation of junctional complexes was evaluated by TEER and paracellular permeability. The TEER value of mouse IEC monolayer gradually increased with culture time, although it was lower than some IECs, such as Caco-2 (14). The paracellular permeability of the cell monolayer was about 3.3% on day 2 of culture and decreased to 0.8% on day 10. These data suggested that mouse IECs were able to establish tight adherent junctions, which could potentially act as an *in vitro* biological barrier. In addition, up to now, the mouse IECs have been maintained for 56 passages. No obvious morphological changes have been observed, and they still possess a normal mouse karyotype. Previous data have also shown that the IEC-6 cells possess a normal rat karyotype, even after 6 month in culture (36). Thus, the mouse IEC line could be used as an *in vitro* model for immunological or toxicological studies at least until passage 56.

Finally, an *in vitro* inflammation assay was performed to determine if the mouse IECs could react to a well-known inflammatory stimulus. IL-1β is known to attract and activate macrophages, natural killer cells, and B and T cells. IL-8 is one of the chemokines that are potent chemoattractants and activators of neutrophils, and it can also attract T cells and monocytes via degranulation of neutrophils (37). IL-6 acts as both a pro-inflammatory and anti-inflammatory cytokine. Previous data have shown that the IECs may be an important source of IL-6 to enhance local mucosal IgA^+^ B cell responses (38). MCP-1, also referred to as CCL-2, is a cytokine from the chemokine family. It can recruit monocytes, memory T cells, and dendritic cells to the tissue injuries or inflammation sites (39). In this study, the stimulated mouse IECs could secrete high levels of IL-1β, IL-6, IL-8, and MCP-1. These four cytokines were also detected in the supernatant of the unstimulated IECs. These findings indicate that the primary mouse IECs can form a cellular barrier, have ability to produce a variety of chemokines and proinflammatory cytokines, and can respond to bacterial infections. Although the detection of these cytokines is not enough to represent the complexity of intestinal mucosal inflammation, the ability of our cultured mouse IECs to secret IL-1β, IL-6, IL-8, and MCP-1 makes it possible to use them in other more complex inflammation assays.

In general, we established a new method for isolation and culture of primary IECs from fetal mouse intestines. A large amount of viable intestinal crypts were obtained using type I collagenase and hyaluronidase. The established cell line had the morphological and immunological characteristics of IECs. Further studies will follow in which the metabolic capacity of this cell culture system will be examined. When this information is completely available, mouse IECs will be a useful *in vitro* model for the study of interactions between pathogen and host enterocytes, and the development of drug delivery systems through the intestinal epithelium.
