# Erratum for: Primary cultures of mouse small intestinal epithelial cells using the dissociating enzyme type I collagenase and hyaluronidase

**DOI:** 10.1590/1414-431X20175831erratum

**Published:** 2017-07-03

**Erratum for:** Braz J Med Biol Res | doi: 10.1590/1414-431X20175831

The Journal would like to correct the order of the authors for correspondence in the article "Primary cultures of mouse small intestinal epithelial cells using the dissociating enzyme type I collagenase and hyaluronidase" that was published incorrectly in volume 50 no. 5 (2017) in the *Brazilian Journal of Medical and Biological Research* <http://dx.doi.org/10.1590/1414-431X20175831>

The correct order of the authors for correspondence is:

Correspondence: L. Ming: <mingliang_2015@sina.com> | J. Cui: <cuij@zzu.edu.cn>

